# Epock: rapid analysis of protein pocket dynamics

**DOI:** 10.1093/bioinformatics/btu822

**Published:** 2014-12-12

**Authors:** Benoist Laurent, Matthieu Chavent, Tristan Cragnolini, Anna Caroline E. Dahl, Samuela Pasquali, Philippe Derreumaux, Mark S.P. Sansom, Marc Baaden

**Affiliations:** ^1^Laboratoire de Biochimie Théorique, CNRS, UPR9080, Univ Paris Diderot, Sorbonne Paris Cité, F-75005 Paris, France and ^2^Structural Bioinformatics and Computational Biochemistry Unit, Department of Biochemistry, University of Oxford, Oxford OX1 3QU, UK

## Abstract

**Summary:** The volume of an internal protein pocket is fundamental to ligand accessibility. Few programs that compute such volumes manage dynamic data from molecular dynamics (MD) simulations. Limited performance often prohibits analysis of large datasets. We present Epock, an efficient command-line tool that calculates pocket volumes from MD trajectories. A plugin for the VMD program provides a graphical user interface to facilitate input creation, run Epock and analyse the results.

**Availability and implementation:** Epock C++ source code, Python analysis scripts, VMD Tcl plugin, documentation and installation instructions are freely available at http://epock.bitbucket.org.

**Contact:** benoist.laurent@gmail.com or baaden@smplinux.de

**Supplementary information:** Supplementary data are available at *Bioinformatics* online.

## 1 Introduction

In drug design, the characterization of binding pockets is a key issue often addressed using molecular dynamics (MD). MD simulations unveil the evolution of large and complex biomolecular systems over time. With increasing computer power, MD generates increasingly large datasets with more frames and particles. The majority of existing programs are not optimized for such large trajectories, so analysis of pocket evolution requires improved software tools that are able to process pocket data in a reasonable time. To tackle this issue, we developed Epock, a software for efficient tracking of protein pocket volume throughout MD trajectories. We demonstrate Epock’s functionality on the ligand-gated *Gloeobacter violaceus* Ion Channel (GLIC) homologue of the human nicotinic receptor (Laurent *et al.*, 2013) and Heat Shock Protein 90 (HSP90).

## 2 Epock program features

**Fig. 1.**
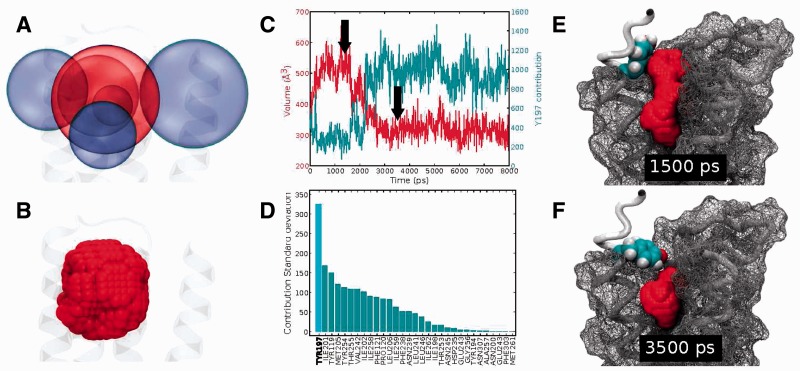
(**A**) Graphical interface of Epock’s VMD plugin for defining the MER using a combination of sphere volumes to include (central sphere) and exclude (surrounding spheres), resulting in a fine-tunable geometric shape for analysis. (**B**) Grid points composing the MER. (**C**) Pocket volume (marked by two arrows) and residue contribution (second curve) of Y197 during an MD simulation. (**D**) Standard deviations of residue contribution ordered from highest to lowest. (**E, F**) Protein conformation (surface in mesh, backbone as tube) and pocket (Y197 as spheres coloured by atom type, pocket accessible space as joint spheres) at *t* = 1500 ps (E) and *t* = 3500 ps (F)

The Epock program takes a topology and MD trajectory as input. A configuration file specifies parameters for each cavity to be characterized, including a maximum encompassing region (MER). The MER provides spatial bounds for each cavity using a combination of simple three-dimensional objects (spheres, cylinders and cuboids) to determine a complex final shape. Epock is therefore intended to follow a priori determined cavities over time, not for cavity identification. Our implementation extends the method proposed by Durrant *et al.* (2011) in the POVME program. The Epock Tcl/Tk plugin for VMD (Humphrey *et al.*, 1996) offers an intuitive way to choose and position shapes to define the MER (see Fig. 1A and B). The Epock command-line tool natively reads xtc trajectory format used by GROMACS (www.gromacs.org). Using the VMD plugin, a variety of ∼13 input trajectory formats can be processed with Epock.

For each pocket, Epock calculates the space accessible to a probe, called ‘free space’, which is the set of all grid points where distance to protein exceeds the user-defined probe radius (typically, 1.4 Å). The number of grid points that overlap each cavity residue is stored and can be outputted as ‘residue contribution’. The volume of the free space is then calculated at higher precision using a finer grid.

In addition to pocket volumes, Epock outputs pore profiles by calculating the radius of the largest disc that fits the previously detected free space along the *Z* axis. Epock produces similar results as the Hole software frequently used to characterize macromolecular channels (Smart *et al*., 1993; Supplementary Fig. S1).

Epock output files include the computed trajectory of free space over time, a feature inspired by the trj_cavity software (Paramo *et al*., 2014). This trajectory is readable by VMD to accessibly illustrate the relationship between pocket volume and protein conformation. Results for pocket volume, residue contribution and pore profile can be plotted in VMD using our plugin or the provided Python scripts (see online documentation: http://epock.bitbucket.org/docs).

## 3 Applications: GLIC and HSP90 binding pocket characterization

The GLIC channel binds general anaesthetics. It is a challenging test case because of its size, 1555 residues and the presence of numerous interconnected pockets. The volume of a single pocket was computed over an 800-frame trajectory of the protein (25 420 atoms, 75 MB).

Epock results are shown in Figure 1. A dramatic decrease of cavity volume is observed after ∼1500 ps (Fig. 1C), alongside particularly high variability in volume contribution of residue Y197 (Fig. 1C and D). Simultaneous visualization of the protein trajectory alongside the pocket free space (Fig. 1E and F) confirms that Y197 side chain movement governs this pocket volume decrease.

We compared Epock performances and validated results with respect to the programs POVME (Durrant *et al*., 2011) and MDpocket (Schmidtke *et al*., 2011) (details in Supplementary Discussion). Epock was the fastest program tested, analysing the 800-frame GLIC trajectory in 6 s. While absolute pocket volumes may differ among programs, volume variations over time are strongly correlated indicating qualitatively identical results (see Supplementary Fig. S2).

In a second application, we used Epock to analyse the volume of the ligand binding domain of the HSP90 based on a range of different conformations and complexes captured by crystallography (as discussed in the original MDpocket paper Schmidtke *et al*., 2011, see Supplementary Discussion and Supplementary Fig. S4). Epock processed this dataset of 86 X-ray structures in PDB format in a matter of seconds, producing output that allowed correlation between the pocket volume and movements of a particular helix formed by residues 100–110, as reported by Wright *et al*. (2004). Analysis highlighted opening of the binding pocket and identified residues implicated in volume variations of up to 200 Å. All observations are in very good agreement with the published results.

All datasets used for benchmarking Epock are available to download from our website.

## 4 Conclusion

To advance our understanding of the events which modulate pocket volume, we present the open-source package Epock, which specializes in rapid pocket volume evaluation for MD simulations. Epock provides detailed output of pocket volume variations, residue contributions and clear visualization of pocket dynamics in VMD.

## Supplementary Material

Supplementary Data
