# Supplementary material for: Epock: rapid analysis of protein pocket dynamics
Source: Bioinformatics. 2014 Dec 12;31(9):1478–80. doi: 10.1093/bioinformatics/btu822 (PMC4410650; doi:10.1093/bioinformatics/btu822)
Supplement: Supplementary Data [file supp_31_9_1478__index.html]

Epock: rapid analysis of protein pocket dynamics — Epock: rapid analysis of protein pocket dynamics — Epock: rapid analysis of protein pocket dynamics — Supplementary Data 

# Epock: rapid analysis of protein pocket dynamics

## Supplementary Data

files

**Files in this Data Supplement:**

- Supplementary Data - pdf file
