# Supplementary material for: Epock: rapid analysis of protein pocket dynamics
Source: Bioinformatics. 2014 Dec 12;31(9):1478–80. doi: 10.1093/bioinformatics/btu822 (PMC4410650; doi:10.1093/bioinformatics/btu822)
Supplement: Supplementary Data [file supp_btu822_SupplementaryMaterial.pdf]

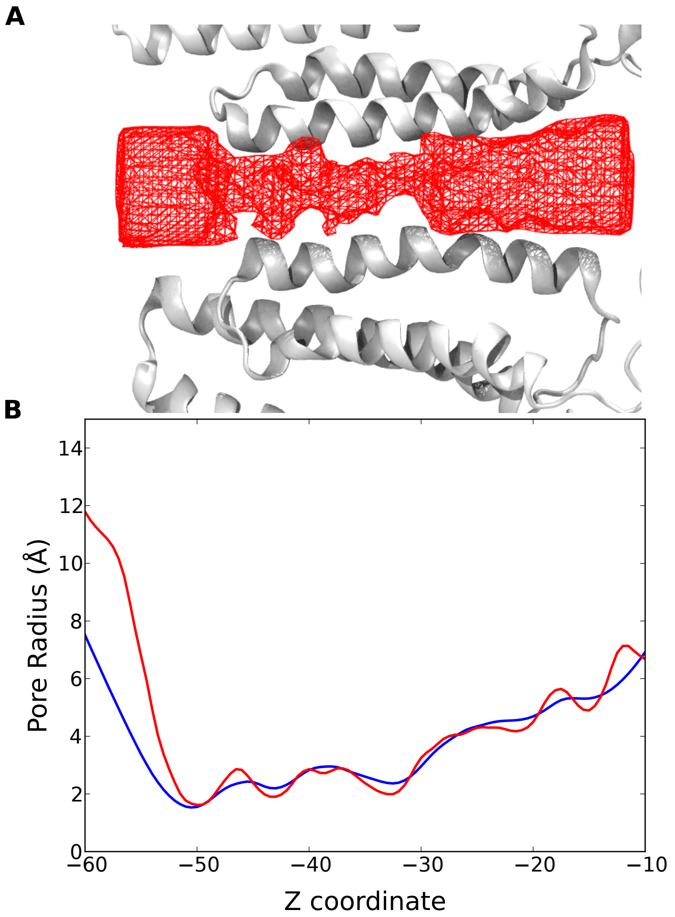

Supplementary Figure 1. A) GLIC transmembrane domain (protein backbone is represented as white cartoon, Epock's pore surface as red wireframe). The pore profile has been calculated given a 14 Å-radius cylinder as include region and a superimposed 7 Å-radius cylinder as contiguous seed (see Epock's manual for more information). The surface has been calculated with the VMD Volmap tool from Epock's output. B) Comparison of the average profile of the GLIC pore of a 800-frame trajectory. Epock (red) and Hole (blue) yield very similar pore profiles.

Supplementary discussion.

Comparison between Epock, POVME and MDpocket.

We benchmarked Epock, POVME and MDpocket programs. POVME (Durrant, de Oliveira, and McCammon 2011) implements an algorithm similar to Epock for free space detection but with a simpler algorithm for free space volume calculation. MDpocket (Schmidtke et al. 2011) uses Voronoi diagrams and has been specifically designed for both transient cavity detection and efficient cavity characterisation. MDpocket computes, in addition to the pocket volume, several other descriptors such as the pocket drugability and hydrophobicity, among others. While this program's goal is clearly broader than simple volume calculation, many authors mention the use of MDpocket to trace single protein pocket volumes along MD trajectories, faced with the lack of software tools able to perform this task. MDpocket can be seen as the current de-facto standard tool for this purpose. Importantly, all three programs can be given the same input grid, allowing for meaningful performance comparisons. In our comparisons we explicitly disabled the search part where MDpocket searches for all the pockets and cavities that can be found among the multiple snapshots collected from a trajectory.

We benchmarked all three programs on the GLIC test case presented in the main manuscript. The volume of a single pocket was computed over an 800-frame trajectory of the protein (25385 atoms, 75 MB) on Mac OS 10.6.8 with 2 × 2.93 GHz Quad-Core Intel Xeon processors and 8 GB 1066 MHz DDR3 memory. The version of the programs used for this benchmark is summarised in Supplementary Table 1.

Epock ran in 6 seconds (i.e less than 0.01 s/frame). This is a dramatically higher speed than both MDpocket and POVME that feature computing times on the hour timescale (9 and 3 hours, respectively). We hypothesise that POVME's execution time is largely related to its implementation in Python, which is known for being slower than a corresponding C++ executable in numerous cases. As MDpocket implements on one side totally different algorithms than Epock, and, on the other side, has been designed to calculate several other descriptors in addition to pocket volume, we investigated the reasons for this slower execution time. Our analysis revealed that, in our example, an important part of MDpocket's execution time is spent in the free space detection part of the program (≈ 37 %, i.e ≈ 16 s/frame), including Voronoi vertex calculation, pocket clustering, and, most importantly, clustering refinement (see Supplementary Figure 3). Importantly, a call to the function "set_pocket_descriptors" represents a significant part of MDpocket's execution time (≈ 44 %, i.e ≈ 19 s/frame). Our investigations revealed that removing the call to this function does not affect MDpocket descriptor output values, at least in our example. Considering this function call redundant, MDpocket's longer execution time may therefore only be marginally due to the calculation of the pocket descriptors it features, which only took up ≈ 3.2 s. Yet, in the current version of the Fpocket suite, MDpocket's execution time is primarily explained by the clustering refinement step and the call to the "set_pocket_descriptors" function. Finally, it is important to note that program performances can be dramatically affected by parameters such as the number of atoms in the systems and the grid size (see Supplementary Table 2).

To complete this benchmark, we compared the three program output volumes. Epock and POVME algorithms for free space detection are very similar. The two programs however drastically differ in their algorithm of free space volume calculation. While POVME uses the same grid for free space detection and volume calculation, Epock uses a second finer grid to calculate the volume, resulting in a more accurate result. Epock has the ability to calculate the free space volume without using a finer grid, hence to calculate the volume in the exact same way as POVME does. This is done by setting the `precision` parameter to 0.0. Jointly with the contiguous point removal disabled, these parameters allow Epock to reproduce POVME's results (Supplementary Figure 2A).

Epock is meant to run with an acceptable degree of precision (precision affects the grid spacing of the volume calculation grid as opposed to the free space detection grid; a precision of 2.0 $Å^{-1}$ corresponds to a grid spacing of 0.5 Å) and possibly with contiguous points removal as well. This set of parameters produces, as expected, more accurate results that are therefore quantitatively different from POVME (Supplementary Figure 2B).

MDpocket features totally different algorithms for both free space detection and volume calculation. As introduced above, MDpocket implements Voronoi diagrams for free space detection. MDpocket then performs several refinement steps before computing the free space volume thanks to a Monte-Carlo algorithm. These elements participate in notable quantitative differences with both Epock and POVME (Supplementary Figure 2B). Importantly, volume variations over time appear statistically significantly correlated (Pearson's correlation test p-values < 10^{-12}), reflecting qualitatively identical results on volume

time evolution for all three programs tested, as depicted by Supplementary Figure 2C showing normalized and smoothed data. It should be noted that by design, Epock slightly underestimates the volume and an estimation of Epock's error bounds is provided on Epock's website. In the example above, Epock's output volume is expected to be less than 10 % below the actual pocket volume that lies within the user-defined maximum encompassing region.

In terms of intended use and efficiency, we would like to highlight that Epock could be a choice program for a user looking primarily for volume tracking along MD trajectories. MDpocket is, to date, the only program to our knowledge able of calculating so many pocket descriptors along MD trajectories. Besides, Epock raw data appears much less noisy than MDpocket, allowing direct analysis of the raw volume data (no smoothing required). Eventually, the data fluctuations may be analysed to detect erroneous conformations along a trajectory that are not representative and may be used as a filter to remove outliers.

Program	Version	Date
Epock	1.0.1rc	2014/09/25
POVME	1.1.0	2014/04/08
MDpocket	2.0	2010/07/15

Supplementary Table 1. Versions and date of the software tools used for comparison in this work. Dates are sources last modification dates according to the software source code hosting service.

Protein	# atoms	# structures	# grid points	Epock	MDpocket	POVME
GLIC	25420	800	7970	00h00m06s	09h10m56s	02h00m30s
HSP90	≈ 1659	86	9771	00h00m06s	00h00m57s	00h01m05s
ER	3988	3036	21247	00h00m41s	02h37m39s	184h04m22s*

Supplementary Table 2. Execution times of Epock, MDpocket and POVME on different systems. Although Epock is consistently the fastest program among the three tested, its run time on the HSP90 system may appear comparatively high with respect to the two other ones. We hypothesise the reason is that, in this case, Epock has to be run through a Python script on each PDB structure while, on the two other systems, the native Epock program ran on Gromacs XTC trajectories. *POVME run time on the ER system has been deduced from its average execution time on 31 frames. The trajectory of the Estrogen Receptor (ER) system is described in Sinha et al., Chembiochem (2010).

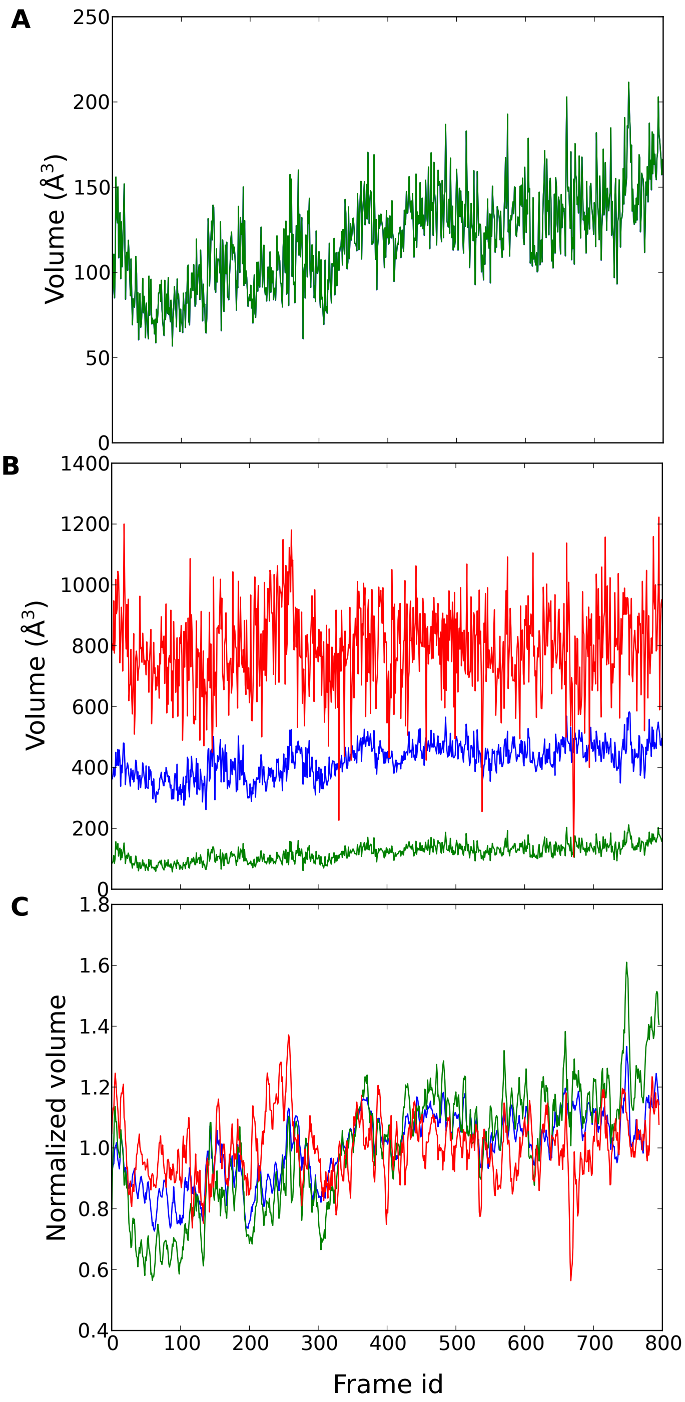

Supplementary Figure 2. Comparison between Epock (blue), POVME (green) and MDpocket (red). A) Comparing POVME and Epock in identical execution conditions with Epock precision parameter set to 0.0 and non-contiguous points removal disabled. B-C) Adding MDpocket to the comparison and setting Epock precision to 2.0. B) Raw data. C) Normalised data (each observed value has been divided by the sample average) and smoothed. The smoothing consists in a moving average over the normalised data with a window size of 5 frames.

```
** LOGGING: Snapshot 3/10
** LOGGING: Opening pdb file...                                          0.01 s.
** LOGGING: Reading pdb file...                                          0.02 s.
** LOGGING: Calculating vertices...                                      1.39 s.
** LOGGING: Clustering pockets...                                        0.96 s.
** LOGGING: Clustering refinement...                                    13.95 s.
** LOGGING: Calculating pocket descriptors...                           19.61 s.
** LOGGING: Drop small and polar pockets...                              2.53 s.
** LOGGING: Sorting pockets...                                           0.00 s.
** LOGGING: Finding vertices of interest...                              1.15 s.
** LOGGING: Allocating memory...                                         0.00 s.
** LOGGING: Writing pqr containing vertices...                           0.01 s.
** LOGGING: Finding atoms that contact the pocket...                     0.00 s.
** LOGGING: Calculating descriptors...                                   3.35 s.
** LOGGING: Volume = 925.43
** LOGGING: Calculating the pocket volume...volume = 947.73 (done in 0.07 s)
** LOGGING: Writing descriptor output file...                            0.00 s.
** LOGGING: Free some memory...                                          0.00 s.
** LOGGING: Free some memory (atom data)...                              0.00 s.
** LOGGING: Free some memory (pocket data)...                            0.86 s.
** LOGGING: Frame done in 43.91 s.
```

Supplementary Figure 3. MDpocket execution profiling. This is an output from our homemade MDpocket executable that allowed us to time code sections at each frame. This is the output for the 3rd snapshot from the GLIC trajectory example.

The example of the Heat Shock Protein 90 (HSP 90)

We have used a dataset of 86 PDB structures of the HSP90 protein determined in different conformations and with different ligands extracted from the list of molecules presented in MDpocket's original paper (Schmidtke et al. 2011). The structures were superimposed using the MultiSeq VMD plugin (Roberts et al. 2006). We defined Epock's maximum encompassing region as an 8 Å radius sphere centred at the centre of mass of residues defined as important in the literature. A smaller sphere has been used as a seed for non-contiguous points removal. The bar chart in Supplementary Figure 3 was obtained by using the Python matplotlib Library (http://matplotlib.org). The rendering of the protein structures and volume of the pocket were performed using the VMD program (Humphrey, Dalke, and Schulten 1996).

We used a Python script to run Epock on the 86 structures of HSP90 (run time of 6 seconds vs 61 seconds for MDpocket). Volume data highlight an opening of the binding pocket, with volumes ranging from 391 Å^3 (PDB id: 3K99) to 572 Å^3 (PDB id: 3R4O). Focusing on structure 3K99, 2 of the 3 main residues highlighted by the Epock contribution analysis (residues N51 and L107) are quoted as key interaction residues in the paper describing this structure (Kung et al. 2010) (see Figure 4 in this paper). The structure 3R4O exhibited the largest volume for the dataset, consistently with the fact that a large compound was co-crystallized in the binding site (Zehnder et al. 2011). The volume increase is related to the movement of a helix formed by the residues 100 to 110 (see central image in Supplementary Figure 4). The importance of this region for the binding of ligands and the evolution of the helix were mentioned by Wright and co-authors (Wright et al. 2004). Again, Epock residue contribution analysis highlights residues that were depicted by the authors as key for the plasticity of this region (N51, M98 and L107).

Thus, without any knowledge of the target structures, the simple calculation of volume and the analysis of residue contributions using Epock allowed highlighting important interacting residues as well as helix movement important for the binding of ligands on HSP90 and thus the design of inhibitors.

All results obtained with Epock as well as the dataset of 86 proteins aligned are available on the Epock website (http://epock.bitbucket.org). This dataset could be useful to perform further analyses for interested readers and allow other researchers to easily compare new methodologies with the Epock implementation.

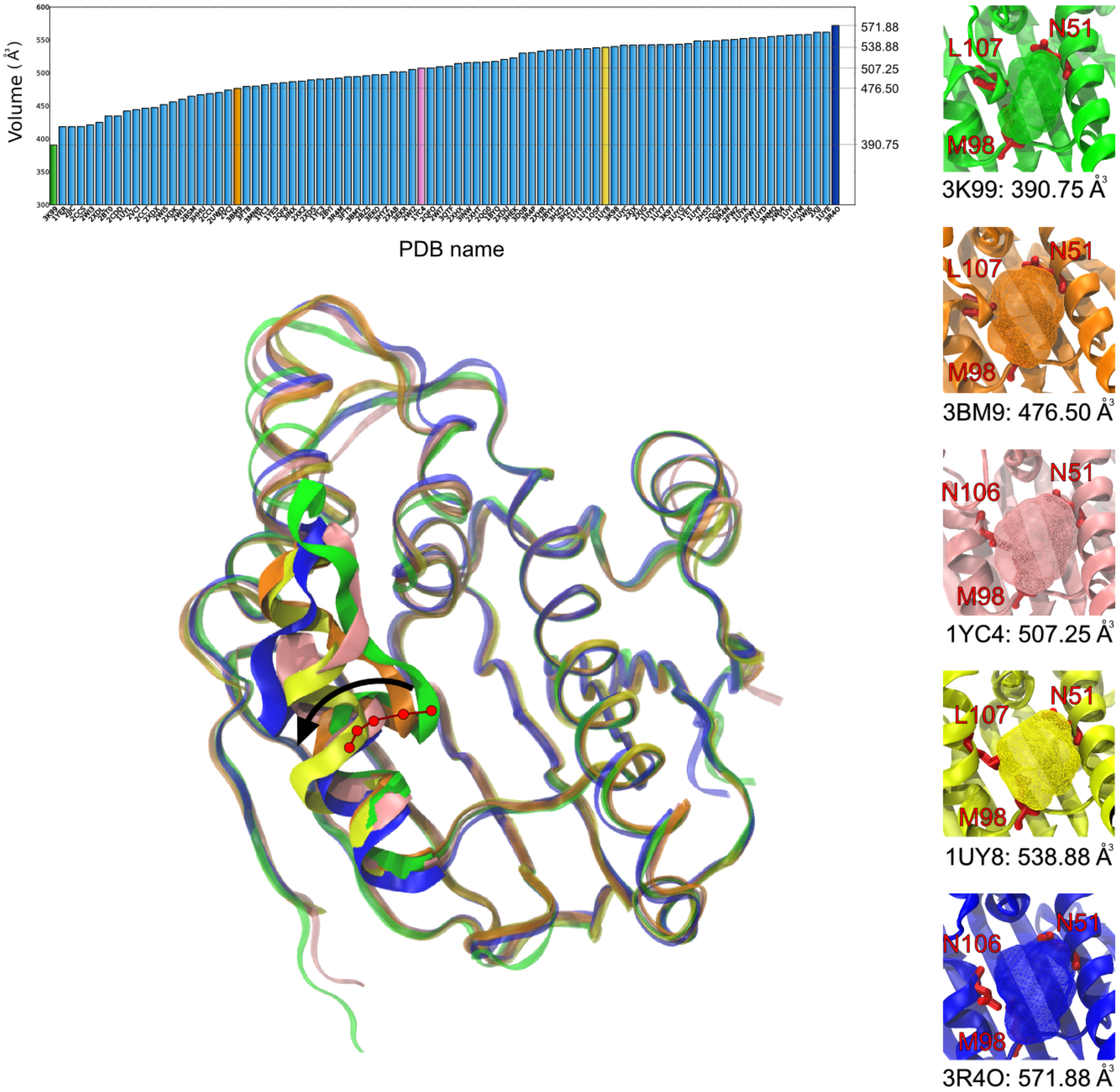

Supplementary Figure 4. Comparison of HSP90 binding pocket volume in 86 X-ray structures. Top panel: barplot showing the pocket volume for each X-ray structure. Selected structures used to illustrate the other panels were highlighted in different colours. Right panel: zoom on the HSP90 binding pocket, highlighting, for each chosen example, the key residues implicated in volume variations. Bottom panel: HSP90 backbone showing the loop movements in each chosen example.

References

Durrant, Jacob D, César Augusto F de Oliveira, and J Andrew McCammon. 2011. "POVME: an Algorithm for Measuring Binding-Pocket Volumes." *Journal of Molecular Graphics & Modelling* 29 (5): 773–776. doi:10.1016/j.jmgm.2010.10.007.

Humphrey, W, A Dalke, and K Schulten. 1996. "VMD: Visual Molecular Dynamics." *Journal of Molecular Graphics* 14 (1) (February 1): 33–8, 27–8.

Kung, Pei-Pei, Buwen Huang, Gang Zhang, Joe Zhongxiang Zhou, Jeff Wang, Jennifer A Digits, Judith Skaptason, et al. 2010. "Dihydroxyphenylisoindoline Amides as Orally Bioavailable Inhibitors of the Heat Shock Protein 90 (Hsp90) Molecular Chaperone.." *Journal of Medicinal Chemistry* 53 (1) (January 14): 499–503. doi:10.1021/jm901209q.

Roberts, Elijah, John Eargle, Dan Wright, and Zaida Luthey-Schulten. 2006. "MultiSeq: Unifying Sequence and Structure Data for Evolutionary Analysis.." *BMC Bioinformatics* 7: 382. doi:10.1186/1471-2105-7-382.

Schmidtke, Peter, Axel Bidon-Chanal, F Javier Luque, and Xavier Barril. 2011. "MDpocket: Open-Source Cavity Detection and Characterization on Molecular Dynamics Trajectories.." *Bioinformatics (Oxford, England)* 27 (23) (December 1): 3276–3285. doi:10.1093/bioinformatics/btr550.

Wright, Lisa, Xavier Barril, Brian Dymock, Louisa Sheridan, Allan Surgenor, Mandy Beswick, Martin Drysdale, et al. 2004. "Structure-Activity Relationships in Purine-Based Inhibitor Binding to HSP90 Isoforms.." *Chemistry & Biology* 11 (6) (June): 775–785. doi:10.1016/j.chembiol.2004.03.033.

Zehnder, Luke, Michael Bennett, Jerry Meng, Buwen Huang, Sacha Ninkovic, Fen Wang, John Braganza, et al. 2011. "Optimization of Potent, Selective, and Orally Bioavailable Pyrrolodinopyrimidine-Containing Inhibitors of Heat Shock Protein 90. Identification of Development Candidate 2-Amino-4-{4-Chloro-2-[2-(4-Fluoro-1H-Pyrazol-1-Yl)Ethoxy]-6-Methylphenyl}-N-(2,2-Difluoropropyl)-5,7-Dihydro-6H-Pyrrolo[3,4-D]Pyrimidine-6-Carboxamide.." *Journal of Medicinal Chemistry* 54 (9) (May 12): 3368–3385. doi:10.1021/jm200128m.
